# Supplementary material for: Light-driven C–H activation mediated by 2D transition metal dichalcogenides
Source: Nat Commun. 2024 Jul 2;15:5546. doi: 10.1038/s41467-024-49783-z (PMC11219765; doi:10.1038/s41467-024-49783-z)
Supplement: Supplementary file 1 — Supplementary Information [file 41467_2024_49783_MOESM1_ESM.pdf]

Supplementary Information

Light-driven C-H activation mediated by 2D transition metal dichalcogenides

Jingang Li et al.

This PDF file includes

Supplementary Figs. 1-14

Supplementary Figures

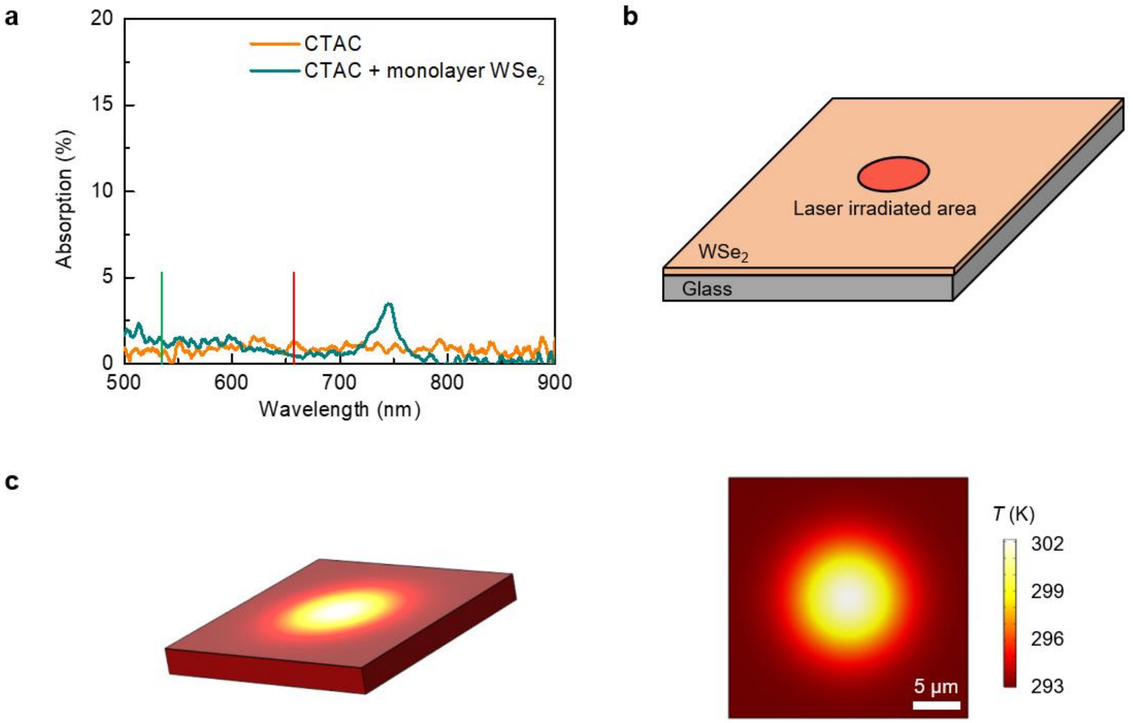

Supplementary Fig. 1. a, Measured optical absorption of CTAC and monolayer WSe_2. The green and red vertical lines indicate the laser wavelengths used in this work, 532 nm and 660 nm, respectively. **b**, Schematic of the simulation setup. **c**, COMSOL simulation of (left) 3D and (right) top-view temperature distribution under 532 nm laser heating. The laser power is 0.2 mW with a beam diameter of 800 nm. Due to the very low optical absorption at the laser wavelength (< 2%), the local temperature increase induced by the laser is less than 10 K.

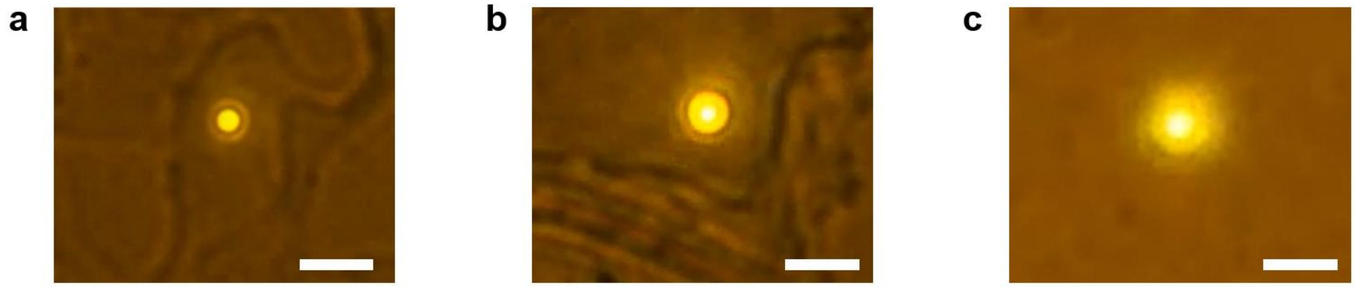

Supplementary Fig. 2. Light-driven synthesis of CDs with larger laser spots. a, ×100 objective; **b**, ×40 objective; **c**, ×20 objective. All scale bars are 5 μm.

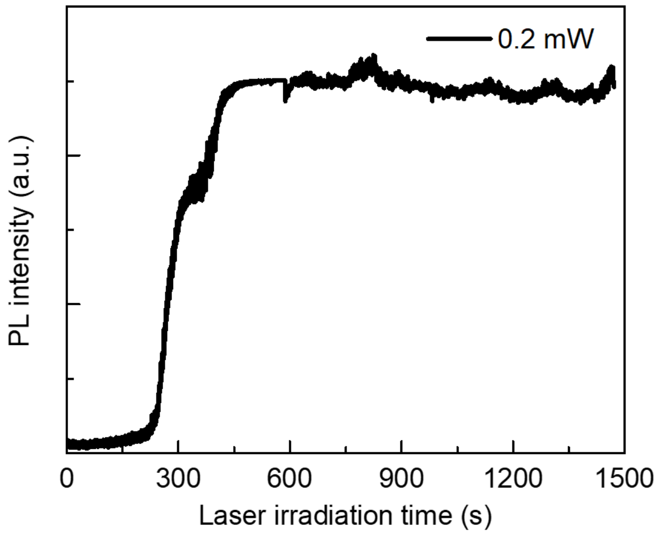

Supplementary Fig. 3. Time-resolved PL intensity of CDs at 600 nm from the CTAC on WSe_2 sample under a 532 nm laser irradiation. The optical power is 0.2 mW. a.u.: arbitrary units.

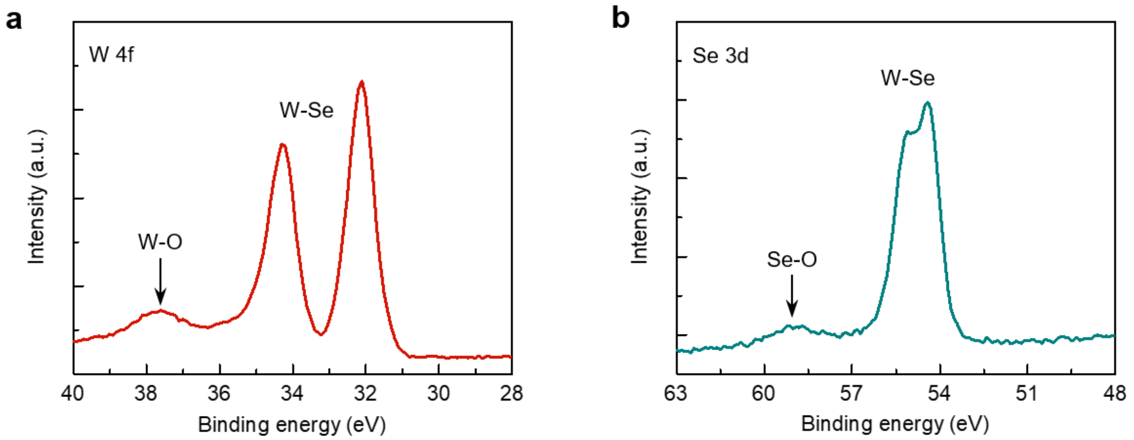

Supplementary Fig. 4. High-resolution X-ray photoelectron spectroscopy (XPS) spectra of W 4f (a) and Se 3d (b) regions of CVD-grown monolayer WSe_2. In addition to the peaks of W $4f_{7/2}$ (~32 eV) and W $4f_{5/2}$ (~34.1 eV) of WSe_2, a small peak at ~37.5 eV can be observed, which corresponds to the W $4f_{5/2}$ from WO_3. Similarly, the appearance of a small peak at ~59 eV for Se 3d suggests the formation of Se-O bonding. The Se vacancies and O adsorption on the surfaces have been regarded as ubiquitous for CVD-grown 2D WSe_2. a.u.: arbitrary units.

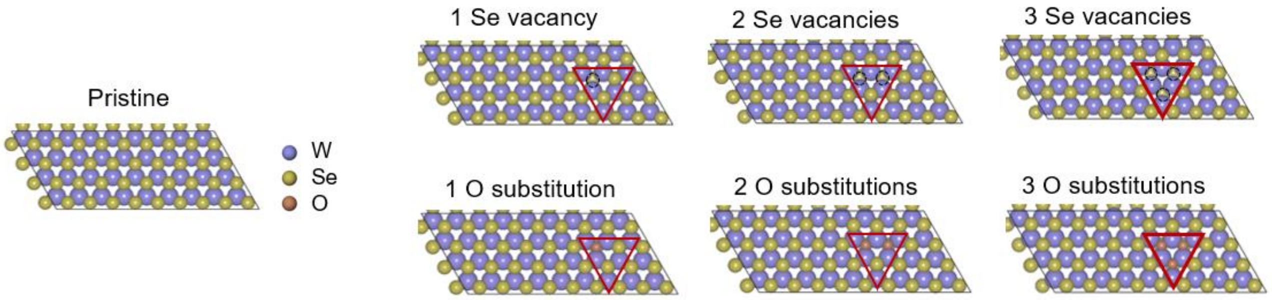

Supplementary Fig. 5. Optimized structures considered for DFT calculations in Fig. 4. Pristine WSe_2 and WSe_{2-x} with Se vacancies or O substitutions are considered. The red triangles highlight the regions with Se vacancies and O substitutions.

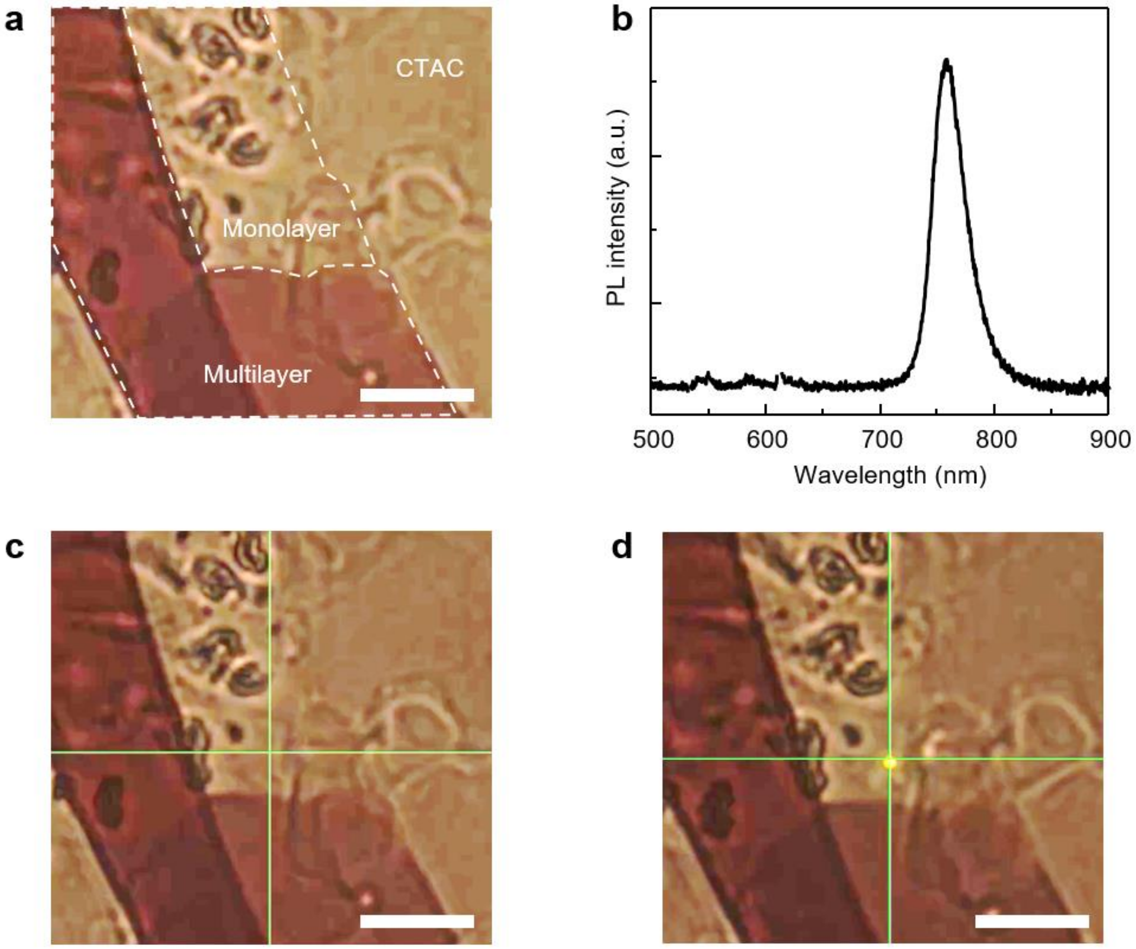

Supplementary Fig. 6. Light-driven C-H activation and carbon dots formation on mechanically exfoliated WSe_2. a, Optical image showing the exfoliated WSe_2 flake coated with a thin layer of CTAC. **b**, Measured photoluminescence (PL) spectrum at the monolayer region. The strong PL mission confirms the monolayer feature. a.u.: arbitrary units. **c**, No obvious PL emission from carbon dots after laser irradiation at 5 mW for 5 min. **d**, The evolution of carbon dots and PL after laser irradiation at 15 mW for 1 min. These results show that a much higher optical power is required for this reaction to occur on exfoliated WSe_2 flake than that on CVD-grown monolayer WSe_2, where the lowest power can be down to 0.2 mW. All scale bars are 10 μm.

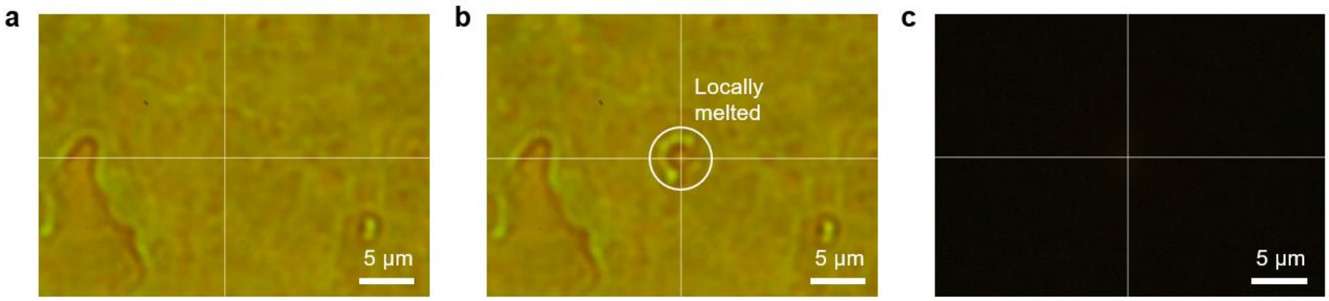

Supplementary Fig. 7. Control experiments on CVD-grown graphene. a, Optical image showing the graphene coated with a thin layer of CTAC before laser irradiation. **b**. After a 10-mW laser irradiation for seconds, the laser heating caused the local melting of the CTAC layer, as indicated in the white circle. The laser position is at the crosshair. **c**, No obvious PL emission from carbon dots in the dark-field image.

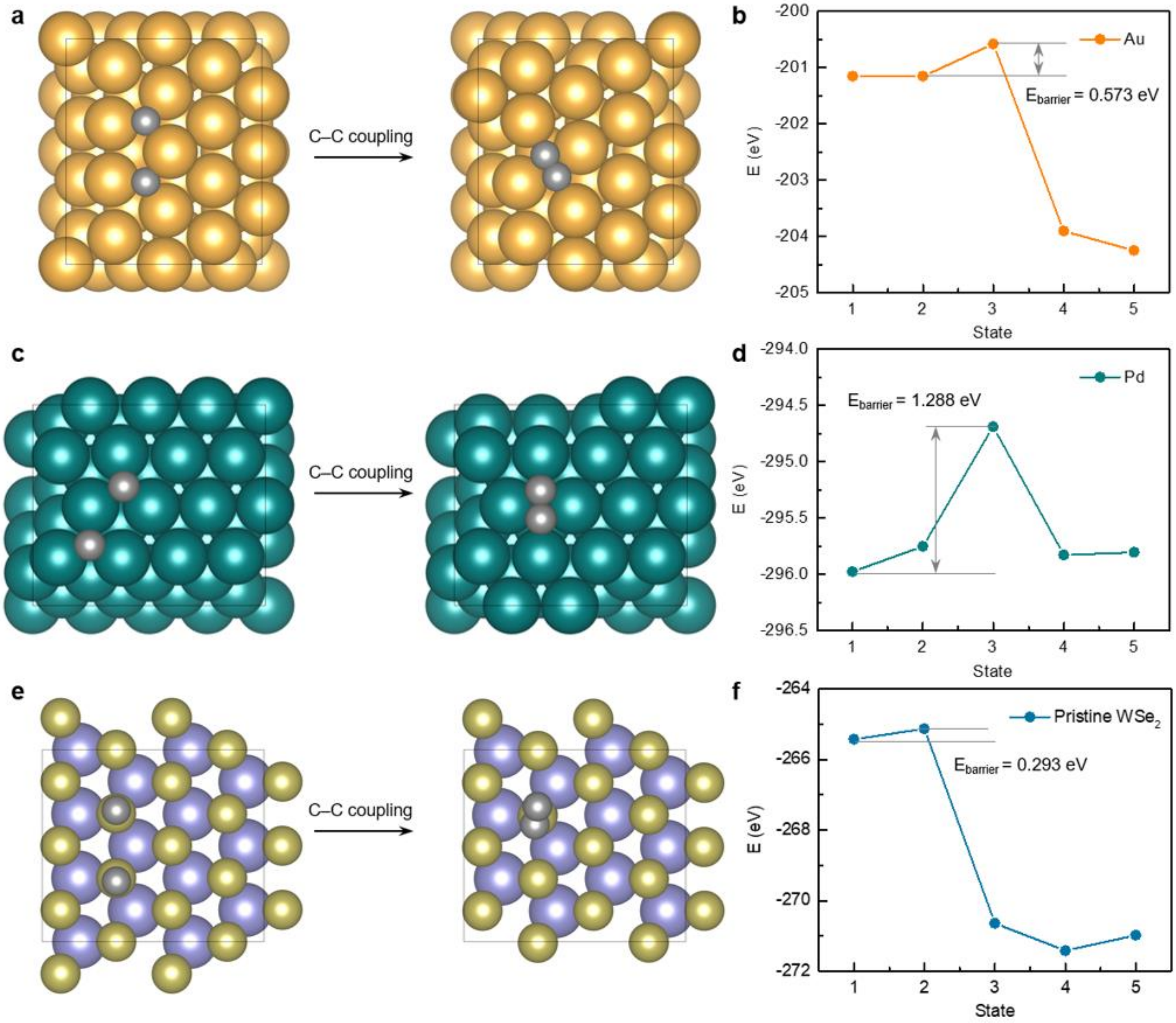

Supplementary Fig. 8. DFT calculations of C-C coupling on different surfaces. a,**c**,**e**, Initial and final states of C-C coupling on (**a**) gold, (**c**) palladium, and (**e**) pristine WSe_2 surfaces. **b**,**d**,**f**, The energy evolution during the C-C coupling on (**b**) gold, (**d**) palladium, and (**f**) pristine WSe_2 surfaces. “1” and “5” denote the initial and final states, respectively. “2-4” are intermediate states.

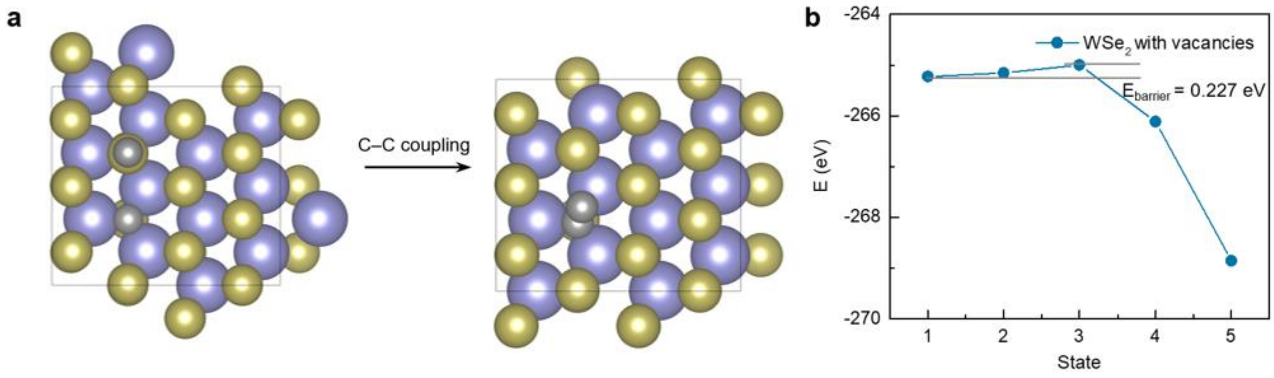

Supplementary Fig. 9. DFT calculations of C-C coupling on WSe_2 surfaces with Se vacancies. (**a**) Initial and final states and (**b**) the energy evolution during the C-C coupling. "1" and "5" denote the initial and final states, respectively. "2-4" are intermediate states.

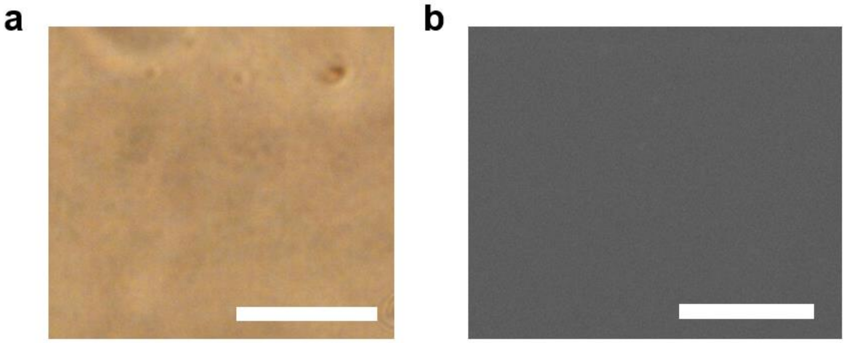

Supplementary Fig. 10. Characterization of the CTAC layer after the laser writing of carbon dots. (**a**) Optical image and (**b**) scanning electron microscopic image of the CTAC layer after the laser writing of carbon dots. The optical power used is 2 mW; the laser wavelength is 532 nm; the laser irradiation time is approximately 10 seconds. Scale bars: 10 μm.

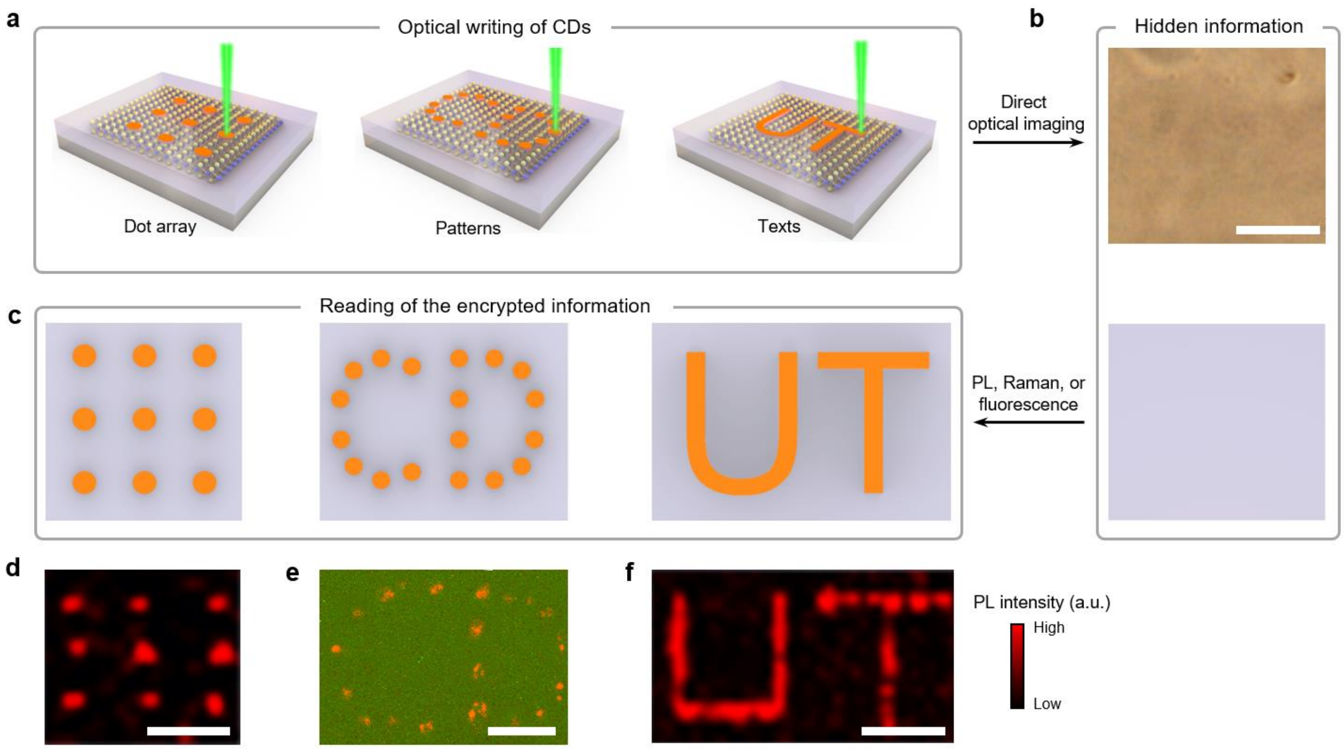

Supplementary Fig. 11. Optically generated CDs for information encryption. a, Schematic showing the optical writing of CDs. **b**, The written CDs remain hidden under direct bright-field optical imaging. **c**, Schematic showing the read-out of the encrypted information by PL, Raman, or fluorescence imaging. **d-f**, PL mapping (**d**), fluorescent imaging (**e**), and Raman mapping (**f**) mapping of the encrypted CDs patterns. All scale bars are 10 μm. a.u.: arbitrary units.

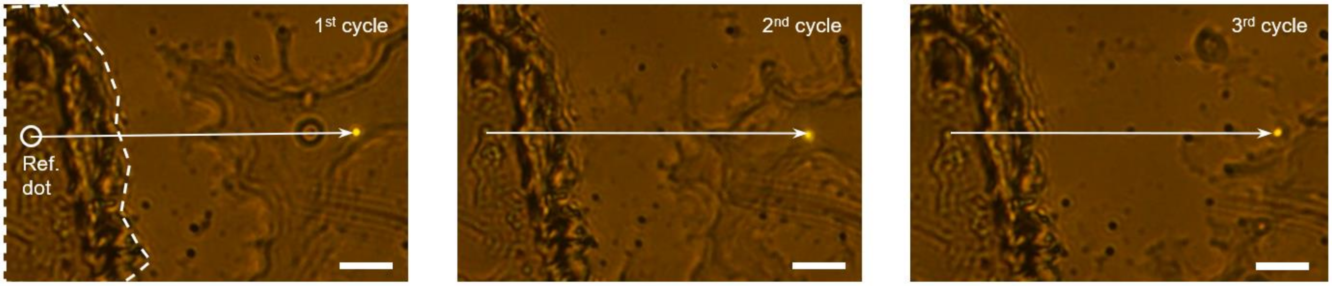

Supplementary Fig. 12. Erasing and rewriting of CDs. CDs were synthesized by laser irradiation on WSe_2 + CTAC sample at the same location three times. The location was marked by the reference scratch/dot and the white arrows. Bright PL emission was clearly observed in all three experiments. All scale bars are 10 µm.

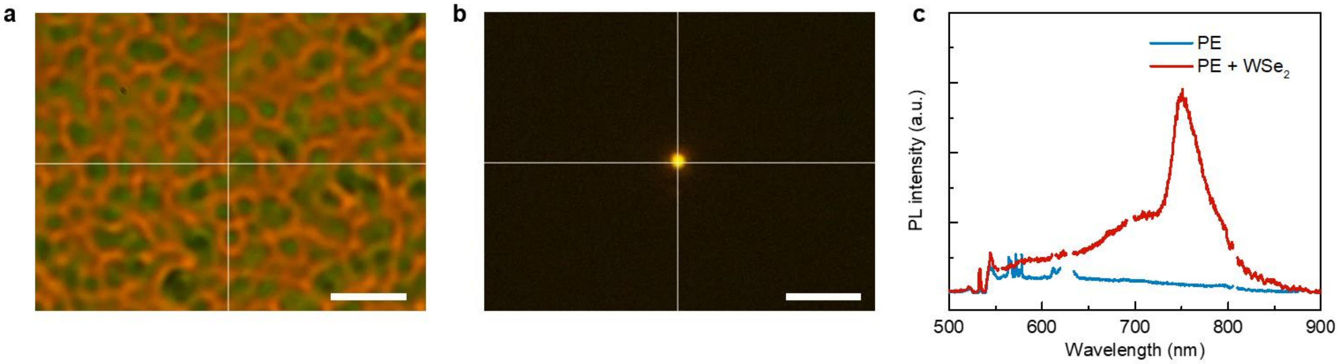

Supplementary Fig. 13. Light-driven C-H activation and CD synthesis with polyethylene + WSe_2. a, Optical imaging of a polyethylene (PE) film on a monolayer WSe_2. **b**, CD synthesis under laser irradiation. Laser power is 3.2 mW. **c**, Measured PL spectra from polyethylene (blue) and polyethylene + WSe_2 sample (red). The latter shows obvious broadband emission band from CDs. Both scale bars are 5 µm. a.u.: arbitrary units.

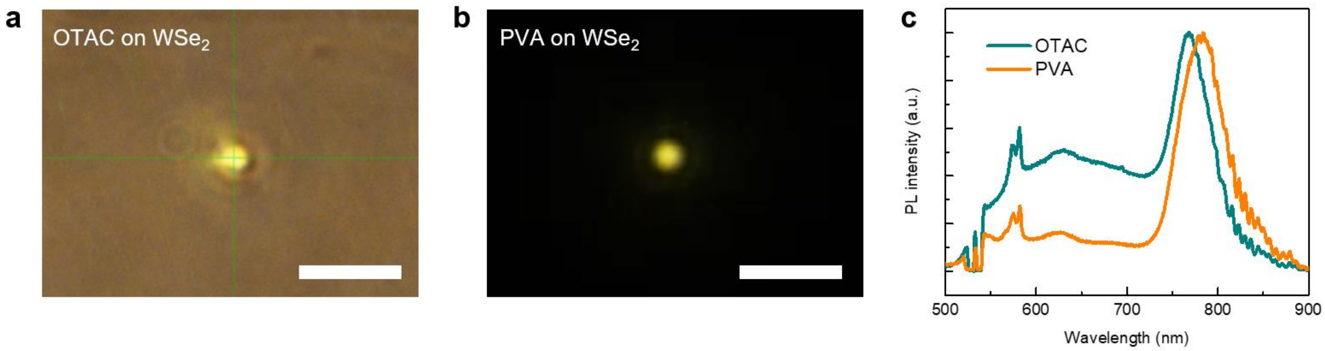

Supplementary Fig. 14. Light-driven C-H activation and carbon dots formation from other organic molecules on a monolayer WSe_2. a,**b**, Optical images showing the PL emission of carbon dots from (**a**) octyltrimethylammonium chloride (OTAC) and (**b**) polyvinyl alcohol (PVA) on monolayer WSe_2. The laser wavelength is 532 nm. Scale bars: 10 μm. **c**, Measured PL spectra from OTAC and PVA on WSe_2 sample, both showing the emission bands from CDs. a.u.: arbitrary units.
