# Supplementary material for: Light-driven C–H activation mediated by 2D transition metal dichalcogenides
Source: Nat Commun. 2024 Jul 2;15:5546. doi: 10.1038/s41467-024-49783-z (PMC11219765; doi:10.1038/s41467-024-49783-z)
Supplement: Supplementary file 3 — Description of Additional Supplementary Files [file 41467_2024_49783_MOESM3_ESM.pdf]

Description of Additional Supplementary Files:

Supplementary Movie 1: PL emission from CTAC on WSe_2 sample under different laser power
